# Giant Coronary-Pulmonary Artery Fistula Incidentally Detected in a Patient Presenting With Acute Inferior Myocardial Infarction

**DOI:** 10.7759/cureus.58627

**Published:** 2024-04-20

**Authors:** Cemre Ozenbas, Abdullah Sukun

**Affiliations:** 1 Radiology, Tınaztepe University Private Buca Hospital, Izmir, TUR; 2 Radiology, Başkent University Alanya Application and Research Center, Antalya, TUR

**Keywords:** myocardial infarction, aneurysm, giant, cta, coronary-pulmonary artery fistulas

## Abstract

Coronary artery fistulas are abnormal connections between the coronary arteries and the heart or other surrounding vascular structures. Although they are usually congenital, they can also occur iatrogenically or due to trauma. They are usually asymptomatic, but they can cause serious and even fatal complications. These complications include myocardial infarction, embolism, thrombosis, arrhythmia, and rupture. In a 54-year-old woman admitted to the emergency department with an acute inferior myocardial infarction, a giant coronary-pulmonary artery fistula was detected on angiography. The fistula could not be closed percutaneously, and computed tomography angiography (CTA) revealed extensive aneurysms and diffuse calcifications. Large fistulas should be closed due to the risk of rupture. Small fistulas should be detected by CTA, and radiologists should be familiar with the imaging features.

## Introduction

The prevalence of congenital coronary-pulmonary artery fistulas (CPAFs) varies between 0.17% and 0.68%, making them the least common type among all coronary artery fistulas, at 15-30% [1,2]. Most CPAFs are classified as anterior type, connecting the proximal part of the coronary arteries to the anterior wall of the pulmonary artery. The co-occurrence of CPAFs and the abnormal origin of the right coronary artery (RCA) from the pulmonary artery (ARCAPA) is referred to as "double trouble." Surgical ligation is recommended in these patients due to coronary steal and myocardial ischemia [3]. In a study analyzing the imaging features of CPAFs, 3975 patients were examined, and a CPAF prevalence of 0.55% (n = 22) was detected. The calculated incidence of aneurysm formation was 59.09%. It was reported that all fistulas resulted from a single drainage area with an average diameter of 2.81 ± 1.48 mm, and the diameter of the fistula with an aneurysm was larger than that without an aneurysm [4]. It has been reported that more than half of the patients with a coronary-pulmonary arteriovenous fistula were asymptomatic, and the fistula connecting the coronary artery to the pulmonary artery could have single or multiple origins. All cases of congenital pulmonary artery fistulas have been reported to drain anteriorly and anterolaterally into the main pulmonary artery and are often accompanied by aneurysmal changes [5]. Although congenital CPAFs are rare, case reports are usually available in the literature. In a systematic review of 103 patients with CPAFs, the mean age was 46 years. The most common associated symptoms were chest pain (39%), dyspnea (25%), and a murmur (37%). The origin of CPAFs was the left main or left anterior descending artery (84%), and the fistula most commonly terminated in the main pulmonary artery (89%). Multiple fistulas were reported in 45% of cases and aneurysms in 19%. Pediatric cases of CPAFs were generally associated with pulmonary atresia and a ventricular septal defect [6].

## Case presentation

**Figure 1:**
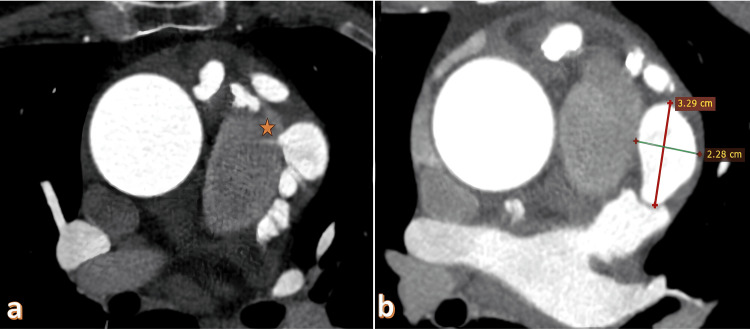
Coronary-pulmonary artery fistula and giant aneurysm. a) Axial coronary CTA shows a fistulous jet flow between the pulmonary artery and aneurysm (asterisk). b) A giant aneurysm measuring 32x22 mm is observed adjacent to the coronary-pulmonary artery fistula. CTA: Computed tomography angiography

**Figure 2:**
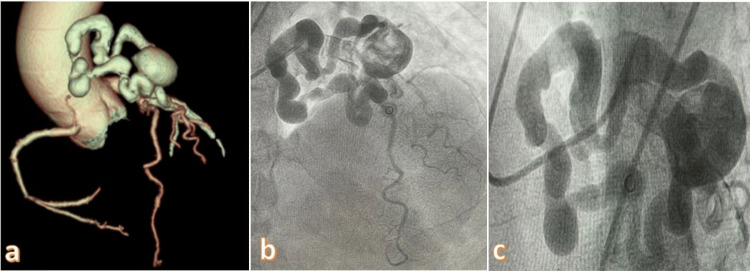
a) Aneurysm and collaterals caused by the coronary-pulmonary artery fistula on coronary CTA 3D imaging. b) Angiogram image of the coronary pulmonary artery fistula. c) Aneurysm and collaterals of the fistula. CTA: Computed tomography angiography

A 54-year-old female patient with known hypertension and type 2 diabetes was referred to the emergency department with a complaint of chest pain. She was diagnosed with an inferior myocardial infarction based on the ECG. Percutaneous coronary intervention (PCI) was performed on the circumflex artery as the CX was 100% occluded in the angiography of the patient. A giant coronary-pulmonary artery fistula was detected in the angiogram. After that, coronary CT angiography was performed to confirm the diagnosis and detail the CPAF structure. A 128-slice multidetector CT scan was performed with a reconstruction interval of 0.625 mm. Following the administration of 90 ml of IV contrast medium at a flow rate of 5 ml/sec with an automatic injector, a coronary artery CT angiogram was acquired. Evaluation was performed by creating orthogonal, oblique, and curved MPR and VR images at the workstation with appropriate software. Curved MPR images of major coronary arteries and 3D VR images of the heart in multiple planes were recorded. The patient has a CPAF originating from the LMA and reaching a diameter of 5.9 mm proximally. The CPAF tract is traced along the left lateral aspect of the main pulmonary artery. A structure is noticed measuring 10.7 mm at its widest point (lumen showing contrast filling) and opening into a saccular aneurysmatic dilatation with a wide neck measuring approximately 32x22x20 mm located in the left lateral aspect of the main pulmonary artery. The right lateral or medial wall of the saccular aneurysmatic dilatation is adjacent to the main pulmonary artery, and the contrast medium has been seen to pass into the main pulmonary artery, consistent with CPAF. 3D-CTA and angiogram images are shown in Figures 1, 2. The patient's native coronary calcium score was 406. The total calcium score with fistula calcifications was 2247. After CTA, PCI was performed again to treat CPAF, but the fistula was not closed due to inadequate access and the risk of rupture.

## Discussion

A CPAF is a rare anomaly where there is an abnormal connection between the coronary artery and the pulmonary artery. In this anomaly, sudden cardiac death may occur depending on the severity of the left-to-right shunt, congestive heart failure, angina, pulmonary hypertension, and aneurysm rupture associated with accompanying aneurysms [7]. We report a case of CPAF in which giant aneurysms, characterized by thrombosis and calcifications, were detected on coronary CT angiography of a patient who remained asymptomatic for years but presented to the emergency department with myocardial infarction.

In the case of CPAF and IGG+4-related disease, CPAF aneurysmal changes were evident. Pericardial nodular inflammation and wall thickening around the left anterior descending artery were reported [8]. In complex CPAF cases, 3D evaluation can be used to assist in preoperative planning and consultation, making the intraoperative management of these patients safer [9]. Coronary artery CTA is a valuable and non-invasive imaging technique for detecting fistulas in the coronary arteries. It plays a crucial role in surgical guidance and identifying small fistulas [10]. The CPAF is much less common in children than adults and small fistulas can be easily missed. Coronary-pulmonary fistulas detected in children are more symptomatic than those in adults. A nine-year-old girl underwent imaging with a chest X-ray, echocardiography, and computed tomography using 3D cinematic imaging. It was found that cinematic rendering images can clearly depict small fistulas [11]. Computed tomography coronary angiography revealed a fistula and pulmonary embolism between the RCA and the coronary sinus in a young patient who complained of left-sided pleuritic chest pain, hemoptysis, and flu-like symptoms. It has been reported that embolism may be caused by turbulent flow and stasis of the CPAF [12].

Antiplatelet and anticoagulant therapies, interventional endovascular procedures, and surgical ligation can be utilized in the management of CPAFs. The aim of endovascular treatment for CPAFs is to destroy the fistula [13]. Percutaneous coil embolization is a minimally invasive and safe treatment method. It is an alternative to complications caused by surgery. The curvature of the developing abnormal vessels necessitates the use of stiffer wires for support, which can complicate the procedure [14]. In our case, the percutaneous method was attempted but was unsuccessful due to widespread aneurysms.

In general, if the aneurysm found in coronary artery fistulas is larger than 30 mm, treatment is recommended due to the significant risk of rupture [15]. In addition to aneurysm size, female gender, saccular aneurysms, and aneurysmal fistulas originating from the left coronary artery are risk factors for coronary artery aneurysm rupture. Surgical ligation can be performed on large aneurysms [16]. The risk factors were present in our patient, but no rupture was detected. This fistula, which was discovered incidentally after the patient presented to the emergency department with acute inferior myocardial infarction, exhibited characteristics that indicated a risk of rupture. The rupture of the CPAF, which is typically detected incidentally, is also spontaneous. In one study, rupture and cardiac tamponade developed as a result of blunt chest trauma [17]. In CPAFs, most patients are asymptomatic and do not require special treatment. However, since it can lead to consequences such as arrhythmia, heart attack, and sudden death, treatment is required in young people and athletes [18].

## Conclusions

The potential problems of CPAFs make it imperative to diagnose the condition and develop a suitable treatment plan. CPAFs can form giant aneurysms and calcifications asymptomatically until advanced age. We can analyze their intricate structures, origins, drainage vessels, and their interactions with the surrounding tissues using CTA and constructed 3D images. It is critical for radiologists to identify patients with CTA prior to rupture, be familiar with their imaging characteristics, and provide therapeutic recommendations.
